# Southern Dental Association

**Published:** 1881-09

**Authors:** 


*Southern Dental Association.*—The annual meeting of this Association was held in the town of Asheville, N. C., commencing July 26, and continuing through four days. The meeting was one of the most interesting it has been the fortune of the writer to attend for some years. Conspicuously was noted the hearty good-will and cordiality of the members present, and their desire to make all present equally welcome. Various papers of interest were read, and the debates over these were lively and animated. The proceedings may be published in future numbers of this Journal. The place of meeting is one of the most picturesque on the Atlantic slope, at an altitude of 2250 feet above tide-water, and a more delightful climate could not be imagined. The pleasant memories of Asheville will long remain.

